# Patient Outcomes after Introduction of Novel Myocardial Protection Protocol for Prolonged Aortic Cross-Clamping

**DOI:** 10.5761/atcs.oa.25-00079

**Published:** 2025-06-12

**Authors:** Masahide Komagamine, Takuma Fukunishi, Yoshiki Yamasaki, Masahiro Tomita, Satoshi Kinebuchi, Daijun Tomimoto, Kan Nawata

**Affiliations:** Department of Cardiovascular Surgery, St. Marianna University School of Medicine, Kawasaki, Kanagawa, Japan

**Keywords:** myocardial protection, clinical outcomes, cardiac surgery, prolonged aortic cross-clamping

## Abstract

**Purpose:** Cardioplegia directly affects patient outcomes after cardiac surgery with prolonged aortic cross-clamping. Our hospital revised its myocardial protection protocol in April 2021 and compared the clinical outcomes of patients with prolonged aortic cross-clamping before versus after the revision.

**Methods:** This study included 36 patients who underwent cardiac surgery via a median sternotomy and prolonged aortic cross-clamping for >4 h at our hospital from 2018 to 2024. Patients treated between 2018 and March 2021 (before the protocol revision) were designated as Group 1, while those treated from April 2021 to 2024 (after the revision) were designated as Group 2.

**Results:** Groups 1 and 2 comprised 17 and 19 patients, respectively. The mean creatine kinase level immediately postoperative was significantly lower in Group 2 versus Group 1 (P = 0.018). The mean hospital stay was also significantly shorter in Group 2 versus Group 1 (P = 0.017). Regarding new postoperative right-ventricular dysfunction, there were 3 cases (15.8%) in Group 2 versus 5 cases (29.4%) in Group 1, but the difference was not statistically significant.

**Conclusion:** These findings suggest that our hospital’s revised myocardial protection protocol, which requires no alteration of the solution itself, achieves safe and favorable surgical results, even in cardiac surgeries requiring prolonged aortic cross-clamping.

## Introduction

In 1964, Bretschneider reported the principle of the Bretschneider solution, an intracellular fluid-type cardioplegia.^1)^ In 1975, Braimbridge, et al. reported the extracellular fluid-type St. Thomas solution No. 1,^2)^ followed by St. Thomas solution No. 2 in 1981, which enabled pH adjustment with a buffer.^3)^ In 1978, blood cardioplegia, in which a potassium compound is added to blood, was reported by Buckberg’s group and has been a superior product with the oxygen-carrying and buffering capacities of blood.^4)^ The antegrade administration of cardioplegic solution to the coronary arteries has been the chosen method from its inception. In 1974, Lolley et al. observed a good myocardial protection effect of retrograde coronary perfusion from the coronary sinus in a study using dog hearts.^5)^

In Japan, many experimental and clinical studies have been conducted since the initial report by Ishizaka in 1977,^6)^ and retrograde coronary perfusion has been widely used in clinical practice since a catheter for perfusion from the coronary sinus was reported by Drinkwater et al. in 1990.^7)^ Methods and protocols for myocardial protection in cardiac surgery are currently left to the discretion of each institution. In particular, in cardiac surgery requiring prolonged aortic cross-clamping, the chosen cardioplegia method directly affects patient outcomes. Therefore, this study details our hospital’s myocardial protection protocol (revised in April 2021) and compares the clinical outcomes of patients who underwent prolonged aortic cross-clamping (>4h) before versus after its revision.

## Materials and Methods

### Target patient group

We included 36 patients who underwent cardiac surgery via median sternotomy at our hospital from 2018 to 2024 and required prolonged aortic cross-clamping (>4 h). Patients treated between 2018 and March 2021 (before the revision) were designated as Group 1, while those treated between April 2021 and 2024 (after the revision) were designated as Group 2. Cases treated emergently with unclear coronary artery evaluation results, those with circulatory arrest, and those treated with coronary artery bypass surgery (including combined surgery) were excluded.

### Cardioplegic solution injection cannula

A 14-G JMS double-lumen CP cannula (JMS Co., Ltd., Hiroshima, Japan) was inserted into the aortic root for antegrade coronary perfusion. A 14-Fr myocardial protection retro self-inflating cannula (Edwards Lifesciences, Irvine, CA, USA) was used for retrograde coronary perfusion. In cases without a right atrial incision, a cannula was inserted into the coronary sinus under echocardiographic guidance. In patients with a right atrial incision, the same cannula was inserted into the coronary sinus opening using a 4-0 polypropylene thread in a purse-string manner and tourniqueted. Pressure was monitored during the administration of the retrograde cardioplegic solution.

### Cardioplegic solution

The composition of the cardioplegic solution was the same before and after the hospital’s protocol revision, and blood cardioplegia (BCP) was used. St. Thomas solution No. 2, a mixture ratio of 4:1 of blood to extracellular fluid, was prepared, and the potassium concentration was adjusted to 20 mEq/L. Before the aortic cross-clamp release, terminal warm BCP (TWBCP) was administered as a general rule (half retrograde, half antegrade).

### Myocardial protection administration protocol

Prior to the protocol change, the cardioplegia administration interval was set at every 30 min, and no fixed rules for antegrade or retrograde administration were observed. Therefore, in many cases, retrograde administration was performed 3 or more times consecutively. In April 2021, the cardioplegia protocol was revised as follows: 1. administration intervals of 25 min; 2. extension of the administration interval (5 min) to once; 3. in principle, antegrade and retrograde drugs were alternately administered; and 4. if alternate administration was not possible, retrograde administration could be performed consecutively once or twice (**Table 1**). At the commencement of antegrade cardioplegia, air was always vented from the side branch while the operator held the right coronary artery ostium with a finger.

**Table 1:** Myocardial protection protocol

	Group 1: Before modification	Group 2: After modification
Administration interval	30 min	25 min
Extension of administration interval (5 min)	Not specified	Up to once
Administration method	Not specified	Alternating antegrade and retrograde (in principle)
Continuous retrograde administration	For cases with 3 or more consecutive administrations	Up to 2 consecutive administrations

### Statistical analysis

The data were analyzed using EZR version 1.28 (Saitama Medical Center, Jichi Medical University, Saitama, Japan), a graphical user interface for R version 3.2.0 (The R Foundation for Statistical Computing, Vienna, Austria).^8)^ Continuous variables are expressed as means ± standard deviation and were compared using the t-test. Categorical variables were evaluated using Fisher’s exact test or the chi-squared test, as appropriate. Statistical significance was set at P <0.05.

## Results

**Table 2** summarizes the patients’ preoperative characteristics. Of the 36 patients, 17 and 19 were in Groups 1 and 2, respectively. The average patient age was 63.5 and 64.5 years in Groups 1 and 2, respectively, and hypertension was significantly more common in the past medical history of those in Group 2 (P = 0.032). As for primary diseases, moderate/severe aortic insufficiency was present in 8 patients (47.0%) in Group 1 and 6 patients (31.6%) in Group 2, versus mitral insufficiency in 7 patients (41.2%) in Group 1 and 12 patients (63.2%) in Group 2. In both groups, more than 80% of the surgical procedures were valve-only, and the simultaneous MAZE procedure was comparable in 5 cases (29.4%) in Group 1 and 5 cases (26.3%) in Group 2.

**Table 2:** Preoperative patient characteristics

	Group 1 (n = 17)	Group 2 (n = 19)	P-value
Age (years)	63.5 ± 16.0	64.5 ± 10.2	0.834
Gender, male, n (%)	10 (58.8)	16 (84.2)	0.139
Body surface area (kg/m^2^)	1.62 ± 0.18	1.71 ± 0.23	0.233
Hypertension, n (%)	8 (47.0)	16 (84.2)	0.032
Diabetes, n (%)	4 (23.5)	3 (15.8)	0.684
Dialysis, n (%)	4 (23.5)	1 (5.3)	0.167
Aortic stenosis moderate/severe, n (%)	5 (29.4)	3 (15.8)	0.434
Aortic regurgitation, moderate/severe, n (%)	8 (47.0)	6 (31.6)	0.495
Mitral stenosis, moderate/severe, n (%)	3 (17.6)	2 (10.5)	0.650
Mitral regurgitation, moderate/severe, n (%)	7 (41.2)	12 (63.2)	0.316
Tricuspid regurgitation, moderate/severe, n (%)	2 (11.8)	4 (21.1)	0.662
Procedure, n (%)			
Valve	15 (88.2)	16 (84.2)	
Valve/aorta	2 (11.8)	3 (15.8)	
MAZE	5 (29.4)	5 (26.3)	

Data are presented as n (%) or as mean ± standard deviation (range).

**Table 3** summarizes the patients’ operative and postoperative outcomes. The average cross-clamping times were 296 and 278 min in Groups 1 and 2, respectively (longer in Group 1); however, there was no significant difference in weaning time from aortic cross-clamp release to cardiopulmonary bypass weaning at 37 and 39 min, respectively (P = 0.734). The creatine kinase (CK) value immediately postoperative was significantly lower in Group 2 (770 ± 382 ng/mL) than in Group 1 (1357 ± 947 ng/mL) (P = 0.018). However, no significant intergroup differences in CK-myocardial band (CK-MB) values were observed. The mean hospital stay was significantly shorter in Group 2 (28.4 days) than in Group 1 (40.4 days) (P = 0.017).

**Table 3:** Operative results and postoperative outcomes

	Group 1 (n = 17)	Group 2 (n = 19)	P-value
Cross-clamp time (min)	296 ± 37	278 ± 39	0.164
Cardiopulmonary bypass time (min)	398 ± 85	383 ± 59	0.553
Weaning time (min)	37 ± 20	39 ± 13	0.734
CK (ng/mL)	1357 ± 947	770 ± 382	0.018
CK-MB (ng/mL)	124 ± 90	82 ± 58	0.105
Extubation (h)	43.3 ± 38.2	35.5 ± 20.1	0.444
Inotropic support (days)	4.9 ± 2.7	3.8 ± 1.6	0.156
ICU stay (days)	6.5 ± 4.8	5.1 ± 1.8	0.263
Hospital stay (days)	40.4 ± 18.6	28.4 ± 8.9	0.017
Morbidity			
Atrial fibrillation, n (%)	11 (64.7)	11 (57.9)	0.742
Surgical site infection, n (%)	1 (5.9)	0 (0)	0.216
Reoperation for bleeding, n (%)	3 (17.6)	2 (10.5)	0.650
In-hospital mortality, n (%)	0 (0)	0 (0)	

Data are presented as n (%) or as mean ± standard deviation (range).

CK: creatine kinase immediately after operation; ICU: intensive care unit; MB: myocardial band

Postoperative atrial fibrillation occurred in more than half of the patients in both groups (n = 11 [64.7%] in Group 1, n = 11 [57.9%] in Group 2); no cases of in-hospital mortality occurred in either group. **Table 4** compares the echocardiographic parameters between groups. There were no significant intergroup differences in preoperative echocardiographic results, left-ventricular end-diastolic diameter, left-ventricular end-systolic diameter, or ejection fraction. Postoperatively, the average left-ventricular ejection fraction was 51.9% in Group 1 and 54.7% in Group 2. Regarding new postoperative right-ventricular dysfunction, there were 3 cases (15.8%) in Group 2 versus 5 cases (29.4%) in Group 1, showing no statistically significant difference.

**Table 4:** Comparison of echocardiography parameters between the 2 groups

	Group 1 (n = 17)	Group 2 (n = 19)	P-value
Preoperative			
LVEDD (mm)	55.4 ± 11.0	53.0 ± 8.2	0.469
LVESD (mm)	37.1 ± 11.4	35.9 ± 8.0	0.722
LVEF (%)	57.8 ± 12.4	59.2 ± 11.5	0.733
Postoperative			
LVEDD (mm)	48.5 ± 7.9	46.7 ± 8.2	0.5
LVESD (mm)	34.6 ± 9.9	31.8 ± 8.7	0.382
LVEF (%)	51.9 ± 16.7	54.7 ± 11.1	0.548
New RV dysfunction, n (%)	5 (29.4)	3 (15.8)	0.434

Data are presented as n (%) or as mean ± standard deviation (range).

LVEDD: left-ventricular end-diastolic diameter; LVESD: left-ventricular end-systolic diameter; LVEF: left-ventricular ejection fraction; RV: right ventricular

## Discussion

Retrospective studies identified a permissible aortic cross-clamping time limit to ensure safe cardiac surgery with myocardial protection. Bezon et al. reported that prolonged aortic cross-clamping cases (mean, 187 min) exceeding 150 min with continuous cold BCP did not demonstrate an increased expected mortality rate.^9)^ Similarly, Nissinen et al. examined the relationship between aortic cross-clamping time and mortality in 3280 adults with continuous BCP and showed that mortality rates increased significantly after durations of 240 min, suggesting that the maximum permissible ischemic time was approximately 240 min.^10)^ The 2024 Guidelines for Myocardial Protection in Open Heart Surgery in Japan describe the maximum permissible aortic cross-clamping time using standard cold BCP as 210–240 min (recommendation class IIa). However, there is currently no established view on the permissible time for aortic cross-clamping before it affects life prognosis and acute surgical results. There is a common conclusion regarding safety at approximately 240 min; however, objective evidence of safety at ≥240 min has not yet been established. In this study, the mean aortic cross-clamping time was 296 min in Group 1 versus 278 min in Group 2, which exceeded the permissible safety time standard of 240 min; however, patients in both groups had good outcomes, with a 0% in-hospital mortality rate. At our hospital, before releasing the aortic cross-clamp, TWBCP is administered as a general rule (half retrograde, half antegrade). TWBCP can reduce myocardial reperfusion injury by enhancing myocardial microcirculation and oxygenation.^11,12)^ Furthermore, it reportedly improves early myocardial contractile function by improving metabolic delay^13)^ and restoring damaged myocardial microtubule structures.^14)^ The use of TWBCP is particularly advantageous in prolonged aortic cross-clamping cases such as those described herein.

Many studies have demonstrated the clinical usefulness of BCP in the selection of a cardioplegic solution for prolonged aortic cross-clamping. The retrospective study by Fedosova et al.^15)^ of crystalloid cardioplegia (CCP) and BCP in adult cardiac surgery demonstrated the validity of the preferential selection of BCP in long-term cases with an ischemic time of ≥211 min. Moreover, the usefulness of BCP for prolonged aortic cross-clamping cases >180 min was also described by Kirklin et al.^16)^ The 2024 Guidelines for Myocardial Protection in Open Heart Surgery in Japan also recommend using cold BCP in cases in which the aortic cross-clamping time is expected to exceed 180 min (recommendation class 1). The advantages of retrograde coronary perfusion include the fact that coronary perfusion can be performed in the peripheral region of the occluded site, even in cases of coronary artery stenosis lesions and aortic valve insufficiency. However, the greatest advantage for surgeons is that coronary perfusion can be performed without interrupting the intraoperative surgical procedure regardless of whether an ascending aortic incision is used. Problems with retrograde coronary perfusion include the fact that a considerable portion of the cardioplegic solution does not reach the capillary bed but instead flows into the ventricle owing to a venovenous shunt and Thebesian channel, which tends to reduce the effective injection volume. In a 1995 report by Ardehali et al., approximately 55% of the retrograde coronary perfusion flow perfused the myocardial tissue as capillary nutritive flow.^17)^ Moreover, concerns about right-ventricular protection have been raised because of the possibility of right-ventricular and posterior ventricular septum perfusion insufficiency and uneven distribution of cardioplegic solution owing to cannula positioning and balloon occlusion.^18,19)^ There are also reports showing the superiority of retrograde myocardial protection with a single administration, but many are targeted at coronary artery bypass cases, and the aortic cross-clamp time is almost always within 90 min. Therefore, even in the recommendation class 1 regarding retrograde perfusion in the 2024 Guidelines for Myocardial Protection in Open Heart Surgery in Japan, the intermittent administration of cold BCP or CCP may be performed via only retrograde administration with an aortic cross-clamping time of 90 min, excluding right-ventricular dysfunction. In contrast, 90% of the antegrade-injected cardioplegic solution perfuses the myocardial tissue, and good right-ventricular myocardial protection is expected in the absence of right coronary artery lesions.^20)^

To establish effective myocardial protection, it is important to fully understand the advantages and disadvantages of antegrade versus retrograde administration. The importance of the combined antegrade and retrograde approach^21)^ and its association with favorable clinical outcomes were previously reported.^22)^ Furthermore, a study using 3-dimensional tracking echocardiography to evaluate on-pump cardiac surgery patients before versus after surgery reported that integrated myocardial protection combining the antegrade and retrograde approaches was superior to the antegrade-only method for protecting the left-ventricular free wall and septal regions.^23)^

In prolonged aortic cross-clamping cases exceeding 4 h, as in the present study, surgeons are focused on completing the surgical procedure; thus, there is a tendency to continuously perform retrograde coronary perfusion without changing the surgical field or interrupting the surgical procedure. There is also concern that the retrograde coronary perfusion cannula position may change because of prolonged surgical manipulation. In response to these concerns, our department changed its myocardial protection protocol in April 2021. With protocol changes, the principle of alternating antegrade and retrograde coronary perfusion was adopted. Moreover, even when retrograde coronary perfusion was continued intraoperatively, the maximum number of consecutive retrograde coronary perfusions was set at 2 considering the above-mentioned concerns regarding retrograde coronary perfusion. Regarding the administration interval, a rule was set to prioritize coronary perfusion at a predetermined time even if the intraoperative operation was interrupted. Crucially, our protocol modification did not involve altering the composition of the protective solution itself. This makes our approach a highly reproducible protocol change, applicable across various institutions regardless of their specific cardioplegic solution. As we became accustomed to the new protocol, the team became proficient in planning when to interrupt the surgical procedure and in performing antegrade myocardial protection. Although the number of antegrade myocardial protection procedures has increased, the time to stop the procedure should have increased as well since no significant difference was noted in cardiac arrest time and the surgical procedure content is generally the same. Thus, we conclude that the effects of procedure interruption were minimized by the meticulous allocation of procedure time.

The preoperative patient profiles were equivalent between the 2 groups before and after the protocol change. The immediate postoperative CK level was significantly lower in Group 2 (770 ng/mL) than in Group 1 (1357 ng/mL) (P = 0.018). Although no statistically significant intergroup difference was observed in the CK-MB values, Group 2 showed a lower mean value (82 ng/mL) than Group 1 (124 ng/mL). In addition, postoperative intubation time, catecholamine support time, and intensive care unit length of stay were reduced in Group 2. These findings suggest that the new myocardial protection protocol may contribute to improved outcomes. Moreover, overall hospital stay (days) was significantly shortened in Group 2 (28.4 days) versus Group 1 (40.4 days) (P = 0.017). However, the impact of the coronavirus disease 2019 pandemic on hospital stay lengths in 2020–2023 cannot be excluded.

There is no absolute indicator for evaluating myocardial protective effects. The left-ventricular ejection fraction on intraoperative echocardiography at the time of cardiopulmonary bypass weaning, as well the postoperative CK-MB value, can be investigated; however, these are merely a values derived once the myocardial protection operation is completed. Intraoperatively, proper cardiac arrest is confirmed, but a clear indicator to confirm its protective effect is lacking.

Evaluating postoperative right-ventricular wall motion is difficult because of the complex morphology of the right ventricle. It is generally evaluated by tricuspid annular plane systolic excursion (TAPSE) and right-ventricular fractional area change (FAC) before and after surgery.^24)^ However, right-ventricular wall motion varies by region, and evaluations using multiple cross-sections are difficult. In the present study, in addition to the TAPSE and FAC values, a comprehensive evaluation was performed by an echocardiographer, and a postoperative right-ventricular wall motion decline was defined as new right-ventricular dysfunction. In the postoperative evaluation of new right-ventricular dysfunction, Group 2 included 3 cases (15.8%), while Group 1 included 5 (29.4%). Although no statistically significant differences were observed, good intraoperative right-ventricular protection may be possible even in prolonged occlusion cases by routinizing regular antegrade perfusion using the new myocardial protection protocol.

### Limitations

The present study had some limitations. First, it was a single-center retrospective nonrandomized study with a relatively small number of patients. Second, the possibility that the coronavirus disease 2019 pandemic influenced hospital stay (days) in 2020–2023 cannot be excluded.

## Conclusion

The current study introduced a new protocol that involved reviewing cardioplegia administration intervals and methods. More improvements in each parameter were observed after versus before its introduction. Our myocardial protection protocol suggests that safe and favorable surgical results can be obtained even in cardiac surgeries that require prolonged aortic cross-clamping.

## Declarations

### Ethics approval and consent to participate

Ethical approval for this study was obtained from the Institutional Review Board (IRB) of St. Marianna University School of Medicine under approval number 6787.

### Consent for publication

We have obtained consent for publication.

### Funding

Not applicable.

### Disclosure statement

Not applicable.

### Data availability

Not applicable.

### Authors’ contributions

Dr. Komagamine (First Author): Conceptualization, Investigation, Formal analysis, Data curation, Visualization, Writing – original draft. Dr. Fukunishi (Second Author): Formal analysis, Investigation. Dr. Yamasaki, Dr. Tomita, Dr. Kinebuchi, Dr. Tomimoto: Investigation. Dr. Nawata (Last Author): Supervision, Writing – review & editing. All authors have read and approved the final version of the manuscript.
